# Avian erythrocytes have functional mitochondria, opening novel perspectives for birds as animal models in the study of ageing

**DOI:** 10.1186/1742-9994-10-33

**Published:** 2013-06-08

**Authors:** Antoine Stier, Pierre Bize, Quentin Schull, Joffrey Zoll, François Singh, Bernard Geny, Frédéric Gros, Cathy Royer, Sylvie Massemin, François Criscuolo

**Affiliations:** 1 Institut Pluridisciplinaire Hubert Curien, University of Strasbourg, Strasbourg, France; 2 Département d’Ecologie, Physiologie et Ethologie (DEPE), CNRS UMR7178, 23 rue Becquerel, Strasbourg Cedex 2 67087, France; 3 Department of Ecology and Evolution, University of Lausanne, Biophore, Lausanne-Dorigny 1015, Switzerland; 4 Faculty of Medicine, University of Strasbourg, Strasbourg EA 3072, France; 5 CHRU of Strasbourg, Physiology and Functional Explorations Department, New Civil Hospital, BP 426, Strasbourg 67091, France; 6 Immunologie et Chimie Thérapeutiques UPR 9021, Institut de Biologie Moléculaire et Cellulaire du CNRS, Université de Strasbourg, Strasbourg, France; 7 Plateforme Imagerie in vitro, IFR 37 CNRS, Strasbourg 67084, France

**Keywords:** Red blood cell, Ageing, Mitochondria, ROS, Oxidative stress, Electron transport chain

## Abstract

**Background:**

In contrast to mammalian erythrocytes, which have lost their nucleus and mitochondria during maturation, the erythrocytes of almost all other vertebrate species are nucleated throughout their lifespan. Little research has been done however to test for the presence and functionality of mitochondria in these cells, especially for birds. Here, we investigated those two points in erythrocytes of one common avian model: the zebra finch (*Taeniopygia guttata*).

**Results:**

Transmission electron microscopy showed the presence of mitochondria in erythrocytes of this small passerine bird, especially after removal of haemoglobin interferences. High-resolution respirometry revealed increased or decreased rates of oxygen consumption by erythrocytes in response to the addition of respiratory chain substrates or inhibitors, respectively. Fluorometric assays confirmed the production of mitochondrial superoxide by avian erythrocytes. Interestingly, measurements of plasmatic oxidative markers indicated lower oxidative stress in blood of the zebra finch compared to a size-matched mammalian model, the mouse.

**Conclusions:**

Altogether, those findings demonstrate that avian erythrocytes possess functional mitochondria in terms of respiratory activities and reactive oxygen species (ROS) production. Interestingly, since blood oxidative stress was lower for our avian model compared to a size-matched mammalian, our results also challenge the idea that mitochondrial ROS production could have been one actor leading to this loss during the course of evolution. Opportunities to assess mitochondrial functioning in avian erythrocytes open new perspectives in the use of birds as models for longitudinal studies of ageing via lifelong blood sampling of the same subjects.

## Introduction

Mitochondria are the crossroads of cell life-and-death processes. First, they are essential to fuel life-sustaining metabolic processes via the production of energy as adenosine triphosphate (ATP) during respiration and oxidative phosphorylation (OXPHOS). Second, mitochondria play a key-role in the cell ageing process, with progressive mitochondrial dysfunctions accumulating with age [1]. Among these alterations, increased mitochondrial production of reactive oxygen species (ROS) appear to be important. Mitochondria are a primary source of ROS, which are molecules having crucial physiological functions, like cell signalling and redox state regulation [2]. However, the production of ROS is also thought to have a pro-ageing effect [3,4]. Indeed, when ROS production is exceeding the antioxidant defences and the repairing cell machinery (a situation defined as oxidative stress), oxidative damage accumulate in all cell components [3,4]. Accordingly, oxidative stress is involved in many cellular defects, which in turn can lead to impairment of tissue functioning and organismal death.

Mitochondria are present in most eukaryotic cell types with few remarkable exceptions, such as mammalian erythrocytes which lose their nucleus and mitochondria during erythroblast maturation [5]. Two non-mutually exclusive reasons have been proposed to explain the loss of mitochondria in mature erythrocytes. First, because the main function of erythrocytes is to carry oxygen but mitochondria are oxygen consumers, the loss of mitochondria during maturation should improve oxygen transport. Note that, although mammalian erythrocytes do not produce ATP through OXPHOS, they can rely on glycolysis to fuel their own energy demanding processes [6,7]. Second, the loss of mitochondria might lessen the exposure of mammalian erythrocytes to the potentially deleterious production of mitochondrial ROS [6], with the theoretical benefit of maximizing their lifespan. The potential implication of mitochondrial ROS production in the loss of mitochondria and nucleus throughout evolution for mammals is referred to hereafter as *the mitochondrial stress hypothesis*. Considering oxygen consumption and ROS production as two factors disfavouring the presence of mitochondria within erythrocytes, it would be expected that similar evolutionary pressures would select for the absence of mitochondria in the erythrocytes of all vertebrate species.

Mature erythrocytes of almost all fish, amphibian, reptile and bird species keep their nucleus during maturation (see [8] for exception in some salamander species), but little is known about the presence and functionality of mitochondria in these cells, except for fish. Indeed, fish erythrocytes have been demonstrated to possess the complete cellular machinery with functional ribosomes [9] and mitochondria [10-13], thus allowing protein synthesis and full cellular activity [14]. In amphibians, mature erythrocytes retain occasionally a few mitochondria, which are often of aberrant morphology [15] even if there is some functional evidence of their presence [16]. In reptiles, there is no clear microscopic evidences to suggest mitochondrial presence in erythrocytes but there seems to be some functional arguments supporting such occurrence [17,18]. Studies examining the presence of mitochondria in bird erythrocytes have reached contradictory conclusions, with some reporting these to be present [19-22], while others report these to disappear during cell maturation [23-25]. Interestingly, a recent study has reported the production of mitochondrial superoxide production within mature avian erythrocytes [26], but more expanded experimental investigation of the presence of functional mitochondria in avian erythrocytes is still lacking.

In the present study, we investigated the presence and functionality of mitochondria in erythrocytes of the zebra finch (*Taeniopygia guttata*). We used transmission electron microscopy (TEM) to demonstrate the presence of mitochondria in avian erythrocytes. We then tested whether those mitochondria were functional by analysing their respiratory activity using high-resolution respirometry, in response to diverse mitochondrial substrates and inhibitors. In addition, we checked their production of ROS using fluorometric assays. Throughout these different steps, we carried out measures on whole blood cells of birds, and we ran in parallel the same analyses of whole blood cells of mice as a negative control since mice erythrocytes lack mitochondria (see materials and methods for details). Finally, to explore whether the presence of mitochondria in erythrocytes leads to higher oxidative stress as stated by the *mitochondrial stress hypothesis*, we compared oxidative stress markers in the plasma of adult zebra finch and a size-matched mammalian model, the mouse.

Our study shows that zebra finches erythrocytes possess functional mitochondria, in terms of respiratory activity and ROS production. However, contrary to the expectations of the *mitochondrial stress hypothesis*, we found no evidence that the presence of functional mitochondria within erythrocytes leads to elevated levels of oxidative stress in the blood of zebra finches (compared to mice).

## Results

### Transmission electron microscopy (TEM)

**Figure 1:**
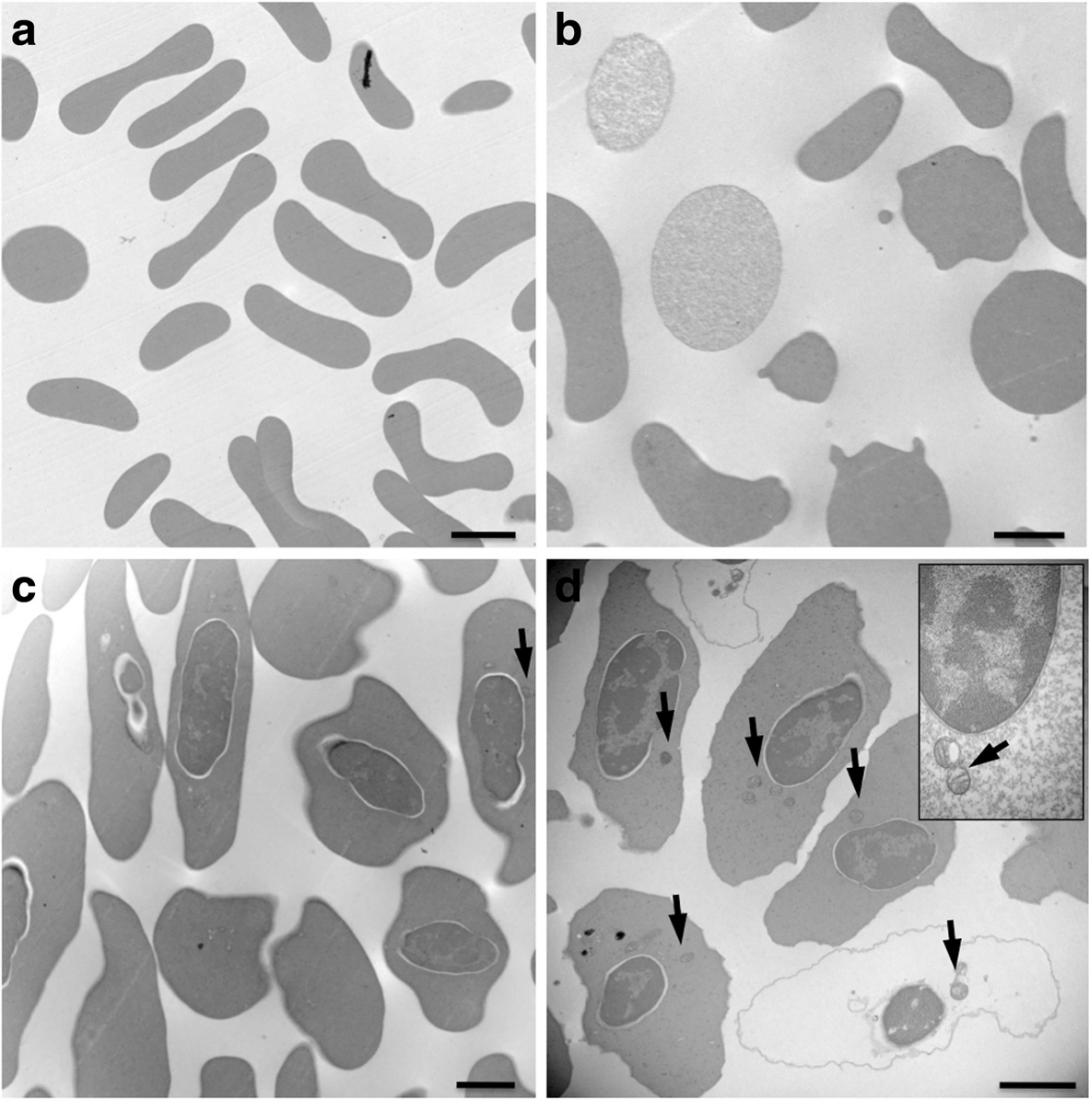
**Transmission electron microscopy (TEM) pictures of mouse (a,b) and zebra finch (c,d) erythrocytes. a**) Standard preparation (PBS) showing mouse erythrocytes with empty cytoplasm. **b**) Saponin treated mouse erythrocytes with empty cytoplasm. Note here that saponin treatment does not seem to deplete completely every cell from haemoglobin content, but alter the cell shape. **c**) Standard preparation showing zebra finch erythrocytes with apparent nucleus and occasional mitochondria (arrow). **d**) Saponin treated zebra finch erythrocytes, with apparent nucleus and numerous apparent mitochondria (arrows). Black bars represent 2 μm.

For mouse, standard TEM preparation (Figure 1a) and haemoglobin-depleted TEM preparation (Figure 1b) did not reveal any cytoplasmic organelles within erythrocytes. In contrast, standard preparation of zebra finch erythrocytes revealed some occasional mitochondria (Figure 1c), while haemoglobin-depleted preparation provided clear evidence of mitochondria in almost every cell (Figure 1d).

### Mitochondrial respiration

**Figure 2:**
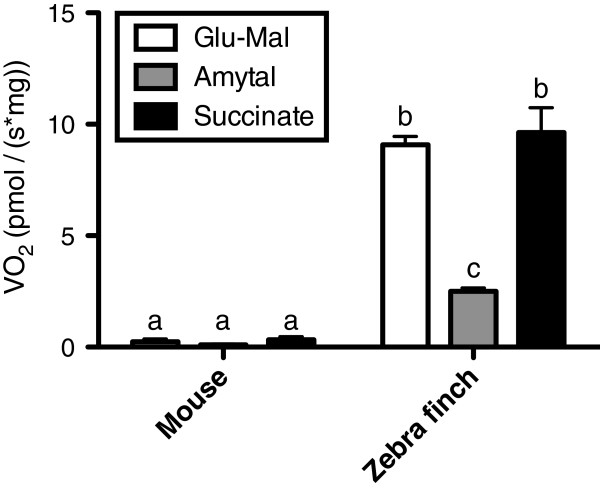
**Mitochondrial respiration rate (VO**_**2**_**) of mouse and zebra finch erythrocytes normalized by the protein content of the cell suspension.** White bars represent baseline respiration rate (on glutamate-malate). Grey bars represent respiration rate after the inhibition of complex I (amytal), which is reversed by the addition of succinate (black bars) in zebra finch. Letters indicate significant differences between groups according to GEE Bonferroni post-hoc (N = 7 per species for each treatment).

Basal rates of oxygen consumption (VO2_glu-mal_) from avian erythrocytes were almost forty fold higher than VO2_glu-mal_ of mammalian erythrocytes (p < 0.001, Table 1, Figure 2). Basal rates of oxygen consumption were significantly different from 0 in zebra finches (one sample *t*-test, *t* = 24.10, df = 6, p < 0.001), but not so in mice (*t* = 1.80, df = 6, p = 0.122). In addition, oxygen consumption was affected by amytal and succinate addition only for avian erythrocytes, as indicated by the significant interaction between species and treatment (p < 0.001, Table 1, Figure 2). Oxygen consumption of mice erythrocytes remains insensitive to the chemical treatments (post-hoc tests: all p-values > 0.5), while in the zebra finch, erythrocytes oxygen consumption decreased following amytal treatment (post-hoc test of VO2_glu-mal_*vs.* VO2_amytal_: p < 0.001), and this inhibition was reversed by succinate addition (post-hoc test of VO2_amytal_*vs.* VO2_succinate_: p < 0.001).

**Table 1:** **GEE model testing species (mouse or zebra finch) and treatment (Glu-mal, amytal or succinate) effects on mitochondrial respiration rate (VO**_**2**_**)**

**VO2 (pmol.s**^**-1**^**.mg**^**-1**^**)**	**Wald *X***^**2**^	**df**	**p-value**
**(Intercept)**	297.1	1	**< 0.001**
**Species**	261.6	1	**< 0.001**
**Treatment**	548.8	2	**< 0.001**
**Species x Treatment**	503.9	2	**< 0.001**

### Mitochondrial ROS production

**Figure 3:**
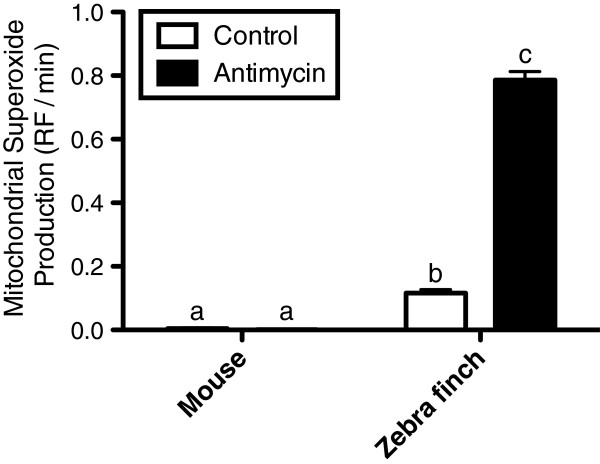
**Mitochondrial superoxide production from mouse and zebra finch erythrocytes, expressed as change in mitosox red® relative fluorescence (RF) per minute.** White bars represent baseline superoxide production and black bars represent superoxide production stimulated by the addition of an inhibitor of complex III (antimycin A). Letters indicate significant differences between groups according to GEE Bonferroni post-hoc (N = 7 per species for each treatment).

Mitochondrial superoxide production was clearly higher in zebra finch than mice erythrocytes (p < 0.001; Table 2, Figure 3). Within-species analyses showed that measures of basal superoxide production were not significantly different from zero in mouse erythrocytes (one sample *t*-test: *t* = 2.39, df = 6, p = 0.054), but significantly greater than zero in zebra finch erythrocytes (*t* = 10.16, df = 6, p < 0.001). Mitochondrial superoxide production was significantly increased by antimycin A treatment in erythrocytes of zebra finches only (post-hoc test: p < 0.001), as indicated by the significant interaction between species and treatment (p < 0.001, Table 2) and illustrated in the Figure 3. Mice superoxide production did not significantly differ between control and treated samples (post-hoc test: p = 0.28).

**Table 2:** GEE model testing species (mouse or zebra finch) and treatment (baseline or antimycin A) effects on mitochondrial superoxide production

***Superoxide production (RF/min)***	**Wald *X***^**2**^	**df**	**p-value**
**(Intercept)**	1151.3	1	**< 0.001**
**Species**	1129.4	1	**< 0.001**
**Treatment**	550.6	1	**< 0.001**
**Species x Treatment**	568.5	1	**< 0.001**

### Plasmatic reactive oxygen metabolites & antioxidant defences

**Figure 4:**
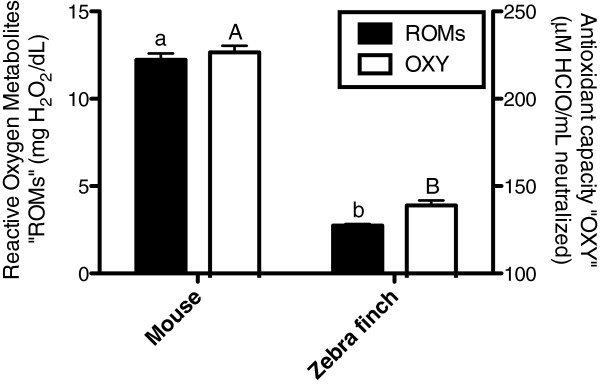
**Plasmatic reactive oxygen metabolites “ROMs” (black bars) and antioxidant capacity “OXY” (white bars) for mouse and zebra finch.** Lower case letters indicate significant differences between groups for ROMs and capital letters for antioxidants levels, according to GLMs (see text for statistics, N = 60 per species).

Reactive oxygen metabolites levels (ROMs) and antioxidant levels (OXY) were higher in plasma of mice compared to zebra finches (ROMs: F = 638.9, df = 1, p < 0.001; OXY: F = 339.3, df = 1, p < 0.001; Figure 4).

## Discussion

The present study shows that mature erythrocytes in the zebra finch retain mitochondria in their cytoplasm. These mitochondria remain functional in terms of respiration, since their rates of oxygen consumption respond to the addition of mitochondrial fuel substrates and respiratory chain inhibitors.

We used transmission electronic microscopy (TEM) to confirm the presence of mitochondria in avian erythrocytes after haemoglobin removal (Figure 1). Some earlier microscopy studies in birds suggested that mitochondria disappear during erythrocyte maturation [23-25], whereas others that removed haemoglobin report mitochondria persistence [19]. Because haemoglobin strongly interferes with light transmission in TEM and because its intra-cellular concentration increases during erythrocyte maturation [27], it is likely that methodological issues account for these discrepancies between TEM studies. Indeed, standard TEM preparations are probably not adapted to detect mitochondria without haemoglobin removal.

We experimentally exposed erythrocytes to inhibitors and substrates known to modulate the activity of the mitochondrial respiratory chain in order to characterize the responsiveness of mitochondrial respiration within zebra finch erythrocytes. Our results show that basal respiration rates of avian erythrocytes were not anecdotal, since oxygen consumption values were similar to those obtained in mouse hippocampal neurons [28]. Overall, our results are in accordance with previous work on fish, indicating that erythrocyte mitochondria are functional in terms of respiratory activities [13]. Together, this challenges a previous conclusion that avian erythrocyte rely only on glycolysis and pentose phosphate pathway [29].

What are the physiological benefits and the associated costs for birds to retain functional mitochondria in their erythrocytes, especially in terms of oxidative stress? Insights on this point should help to understand why mammalian erythrocytes have lost their mitochondria or, alternatively, why birds (and probably other non-mammalians vertebrates [12,16,17]) have retained functional mitochondria within their erythrocytes.

### What physiological role for mitochondria within erythrocytes?

Mitochondrial biogenesis is under the control of nuclear genes [30]. Hence, mammalian erythrocytes might lose their mitochondria due to nucleus extrusion rather than due to selection against the presence of functional mitochondria. A cost of keeping respiration is that to sustain cellular oxygen consumption (Figure 2), avian erythrocytes have to synthetize the electron transport chain proteins. In contrast, containment of greater functional energetic capacity in these cells might allow various cellular activities based on high rate of ATP production through OXPHOS, as demonstrated earlier for fish [11]. Among other things, those activities might include the constant synthesis of various proteins such as haemoglobin or heat shock proteins (HSP). Constant turnover of haemoglobin throughout erythrocyte lifespan might allow the optimization of oxygen binding and transport [11], and HSP protein synthesis might allow stress resistance of erythrocyte metabolism ( [31], and see [14] for HSP implication in stress metabolism in fish erythrocytes). Moreover, functional energetic machinery in these cells could permit other energy-requiring functions such as immune responses [32], as suggested for instance by natural and experimental phagocytosis by erythrocytes in amphibians [33]. All this relies on the assumption that avian erythrocytes are able to sustain protein synthesis, an idea which is not completely supported by earlier studies on transcriptional capacities of avian erythrocytes nuclei (reviewed by [34]). More work is needed to clarify protein synthesis capacities of avian erythrocytes and their potential cellular effects. Indeed, it is not excluded that the persistence of functional mitochondria within avian erythrocytes is associated with net negative rather than positive effects. First, for selection to favour the silencing of mitochondrial biogenesis within avian erythrocytes, those mechanisms should be available for selection to act upon, which still needs to be demonstrated. Furthermore, even if pathways favouring the silencing of mitochondrial biogenesis do exist in avian erythrocytes, those pathways might not be selected if they have antagonistic pleiotropic effects, being advantageous during cell maturation but detrimental later in the cell life.

Of note, besides supporting energetically demanding cellular processes, the synthesis of ATP (achieved by OXPHOS but also potentially by glycolysis) might modulate oxygen transport by haemoglobin. Accordingly, the intra-cellular ratio ADP/ATP could be an important modulator of Hb-O_2_ affinity, since the addition of ATP has been found to increase haemoglobin affinity to oxygen in reptile erythrocytes [17]. Our pilot experiments on birds support the potential importance of the ADP/ATP ratio in oxygen transport regulation, since ADP addition during respiration measurements of avian erythrocytes causes a strong release of O_2_ (Additional file 1: Figure S1). This phenomenon could be interpreted as a decrease of Hb-O_2_ affinity, and should encourage future work on the regulation of Hb-O_2_ affinity by ADP/ATP ratio and mitochondrial activity.

### The mitochondrial stress hypothesis

The unavoidable by-product of OXPHOS is the generation of potentially deleterious ROS [3]. Hence, the *mitochondrial stress hypothesis* argues that mammalian erythrocytes have lost their mitochondria in order to down-regulate cell oxidative stress [6]. Zhang and collaborators [6] have previously demonstrated that ROS production in mammalian erythrocytes remain stable under metabolic circumstances known to induce oxidative stress (i.e. hyperglycaemia, ischaemia), whereas ROS production in avian erythrocytes is dramatically increased under the same conditions. These authors concluded that nuclear and mitochondrial extrusion may help mammal erythrocytes to lower ROS production under metabolic stress, which is in line with the suggested longer lifetime of mammalian erythrocytes compared to birds [35]. Several lines of evidence call into questions the importance of oxidative stress as one major driver of mitochondria loss by mammalian erythrocytes.

Mice exhibited higher plasmatic oxidative stress. Hence, it suggests that even if mice erythrocytes are lacking mitochondria, their immediate environment (i.e. plasma) suffers from greater oxidative damage despite a higher plasma antioxidant capacity than zebra finches (Figure 4). This challenges the *mitochondrial stress hypothesis* proposed by [6], and suggests that the presence of functional mitochondria within avian erythrocytes does not necessarily compromise blood oxidative state. Still, our data do not rule out the possibility that an oxidative imbalance may occur at the scale of the erythrocyte but a complete comparative study is needed to resolve this point. If erythrocytes of birds accumulate oxidative damage at a higher rate because of their mitochondria, we might expect avian erythrocytes to have a shortened lifespan. Mouse erythrocytes turnover seems however to be faster than in chicken, pigeon or duck [36], which is contradictory with [35] assumptions. Because numerous (confounding) parameters might affect erythrocyte lifespan, such as body size/weight [37], further investigations at the inter-specific level are required before firmly concluding on this point. Research focusing at inter-individual variation in cell mitochondrial abundance and oxidative stress should also be encouraged. Here, it is worth mentioning that a few salamander species from five different genera of the subfamily *Bolitoglossinae* show relatively high amounts (> 80%) of enucleated erythrocytes [8]. Using such species could provide new insights on the evolutionary loss of nucleus and mitochondria also observed for mammalian erythrocytes.

Finally, it is well-known that ROS can trigger cell senescence via mitochondrial driven apoptosis and the opening of the mitochondrial permeability transition pore [38,39]. However, previous studies have shown that chicken erythrocyte cell death does not rely on such a caspases’ apoptotic pathway [40]. Therefore, as stated by [13], “mitochondria are probably a minor contributor to oxidative stress in erythrocytes”, and hence mitochondria loss in mammals has probably no or only a minor relationship with a reduction of oxidative stress. Indeed, even if the presence of mitochondria within avian erythrocytes was associated with ROS production (Figure 3), the oxidative imbalance observed in the blood was lower for zebra finch than for mice (Figure 4). Therefore, the presence of mitochondria within erythrocyte does not necessarily seem to be associated with increased levels of oxidative stress, perhaps due to efficient intra-cellular antioxidant defences. This point is further supported by a pilot experimental approach where we tested whether mitochondrial ROS production of avian erythrocytes is increased under hyperglycaemic conditions, as suggested by [6]. In this experiment, mitochondrial superoxide production was not affected by hyperglycaemic conditions (30 mM Glucose, Additional file 1: Figure S2).

### Perspectives

The fact that avian erythrocytes possess functional mitochondria presents research potential both for evolutionary and ageing studies. In the recent past, numerous studies have addressed the implication of oxidative balance in the set-up and evolution of life history trade-offs [41-43]. However, due to practical and ethical constraints, most studies on vertebrates focused on plasmatic parameters to assess “organismal” oxidative stress. The presence of functional mitochondria in non-mammalian (fish, birds) erythrocytes provides a good opportunity to investigate both sides of the oxidative balance (mitochondrial ROS production and antioxidant defences), using only blood samples. Moreover, while mitochondrial research in mammals requires animal culling to collect tissues and extract mitochondria for functional studies, we can now use lifelong blood sampling of the same birds to investigate mitochondria functioning with a longitudinal experimental design. Hence, the use of erythrocytes in non-mammalian vertebrates as a source of mitochondria should be beneficial for ageing studies by providing a more powerful tool than classical cross-sectional studies to investigate mitochondrial role and modifications associated with ageing process and life history traits (such as the uncoupling state of mitochondria [44,45]). It should also help to investigate the implication of mitochondria in ageing rate variability of wild and non-model animals, which are often submitted to restricted ethical rules.

## Conclusion

Our findings demonstrate that avian erythrocytes possess functional mitochondria in terms of respiratory activities and ROS production. Therefore, our results combined with available literature on other vertebrates suggest that mammals are almost unique in having an evolutionary loss of mitochondria by mature erythrocytes. Since mitochondria within avian erythrocytes does not appear to result in plasma-level oxidative stress, our results challenge the idea that mitochondrial ROS production was a major factor leading to this loss during the course of evolution. Finally, the presence of functional mitochondria within avian erythrocytes open new perspectives in the use of birds as models for longitudinal studies of ageing via lifelong blood sampling of the same subjects.

## Materials and methods

### Experimental procedure

The study complied with the ‘Principles of Animal Care’ publication no.86-23, revised 1985 of the National Institute of Health, and with current legislation (L87-848) on animal experimentation in France.

Experiments were realized on adult zebra finches and mice (C57BL/6) coming from our main captive populations. Birds were placed as unisex pair in cages (0.57 × 0.31 × 0.39 m) and provided with food (a commercial mix of seeds for exotic birds enriched with vitamins and eggs) and water *ad libitum*. Birds were housed at 23°C on a 13 L: 11 D light cycle. Mice were placed in small unisex groups (2 to 5) in plastic cages (40 × 25 × 15 cm) with *ad libitum* access to food (SAFE A03) and water. Mice were housed at 25°C on a 12 L: 12 D light cycle. Blood samples (30 to 100 μL depending on the experiment conducted) were collected in heparinised glass capillaries from the brachial vein for the zebra finches and from the submandibular vein for mice. Considering the difficulties to appropriately separate nucleated erythrocytes from white blood cells considering their similar density (personal observation), we choose to use total blood cells of zebra finch, and to add a size-matched mammalian negative control. Indeed, white blood cells represent only a small fraction of whole blood compared to erythrocytes (around 1 white blood cell for 800 red blood cells [46]). Moreover, mammals and birds possess a similar small fraction of white cells in whole blood (total white blood cells counts for: BALBc mice ≈ 10.10^3^/μL [47] and Scarlet Rosefinch ≈ 8.10^3^/μL [46]). Hence, we use the term ‘erythrocytes’ rather than ‘total blood cells’ throughout the text when describing and discussing our results.

### Transmission electron microscopy (TEM)

A 10 μL aliquot of a fresh blood sample was diluted in 390 μL of phosphate buffered saline (PBS) before centrifugation (300 · *g* for 5 · min) to pellet the cells. Cells were subsequently fixed or treated with saponin (200 μg/ml for 15 min at 4°C followed by a centrifugation at 8000 · *g* for 10 · min) before fixation to remove/limit haemoglobin interferences. The cells were fixed with 2.5% glutaraldehyde (Fluka Analytical, Sigma) in 0.1 M phosphate buffer for 24 hours. Cells were post-fixed with 0.1% osmium tetroxyde (EMS) in water for one hour, and dehydrated through a series of ethanol baths before being embedded in araldite M (Fluka Analytical, Sigma). Thin sections were observed under Hitachi H7500.

### Mitochondrial respiration

Fresh blood samples (80 μL) were diluted immediately after collection in 1 mL PBS and kept on ice until analysis. Respiration measurements were made within four hours after blood collection. Before analysis, blood was centrifuged (300 · *g* for 5 · min) to pellet cells and discard the plasma fraction. Cell pellet was then diluted in 2 mL of respiration buffer (CaK2EGTA 2.77 mM, K2EGTA 7.23 mM, MgCl_2_ 6.56 mM, imidazole 20 mM, taurine 20 mM, dithiothreitol 0,5 mM, K-sulfonate methane 50 mM; glutamate 5 mM, malate 2 mM, phosphate 3 mM; 2 mg/ml fatty acid free bovine serum albumin (BSA) and 125 μg/mL saponin, pH 7), and placed into respirometry chamber. Each time one mouse sample (negative control) was run in parallel of one zebra finch sample. Oxygraph-2 k system (O2k, OROBOROS INSTRUMENTS, Innsbruck, Austria) contain two respirometry chambers at 37°C, 750 rpm stirrer speed, and two point calibrations of the OROBO-POS polarographic oxygen sensors. For this experiment, 7 mice and 7 zebra finches were blood sampled, and each blood sample was measured three times consecutively. A first time for the baseline respiration (VO2_glu-mal_) on glutamate-malate, a second time after amytal addition (2 mM; VO2_amytal_), which is an inhibitor of the complex I, and a third time after succinate addition (15 mM; VO2_succinate_), which is a substrate of the complex II. Mitochondrial respiration rates were normalized by protein content of the cell suspension, measured after cell lysis by Pierce BCA protein assay (THERMO scientific). Respiration rates (VO_2_) are expressed in pmol O_2_ consumed.s^-1^.mg protein^-1^.

### Mitochondrial ROS production

For each individual, two 10 μL aliquots of fresh blood were diluted in 390 μL of PBS and stored on ice prior to analyses, which were completed within 3 · hours of sampling. Prior to staining, diluted blood was centrifuged (300 · *g* for 5 · min) to pellet cells and discard the plasma fraction. MitoSOX Red (Molecular Probes, Life Sciences) was then added at a final concentration of 5 μM in each sample. Each individual was measured two times, the first time to determine basal superoxide production, and the second time to assess a potential increase in superoxide production in response to a specific mitochondrial inhibitor. In these latter samples, Antimycin A (a complex III inhibitor, known to increase mitochondrial superoxide production) was added at a final concentration of 100 μM. Cells were subsequently incubated for 25 · min (at 37°C for mice and 40°C for zebra finches), then washed with PBS by centrifugation as described above and held on ice until analysis by flow cytometry. All flow cytometry measurements were performed using a FACScalibur cytometer (BD Bioscience, San Jose, CA) with blue laser-excitation at 488 nm. Emitted fluorescence was collected on the FL2 channel detector (575 ± 13 · nm bandpass filters). A T_0_ acquisition was done for each sample and after 30 min of incubation (37 or 40°C), a second acquisition (T_30_) was made to evaluate the increase in mitochondrial superoxide. Data were acquired and analysed using CellQuest Pro v5.1.1 software (Becton Dickinson). Analyses were performed by gating on live cells to exclude debris and dead cells. Gating was defined according to morphological indications provided by forward and side scatter detectors. For each sample, 20 · 000 events were acquired. Analyses allowed defining the geometric mean of fluorescence (which limits the influence of extreme values). We expressed superoxide production as the increase in relative red fluorescence per minute (RF/min). For this experiment, superoxide production was determined for 7 mice and 7 zebra finches. Preliminary analyses show that flow cytometry measurements of duplicate samples from the same individuals were highly intercorrelated (r = 0.92, p < 0.001, N = 14) and intra-individual variation was low (CV = 6.43%).

### Plasmatic reactive oxygen metabolites & antioxidant defences

The antioxidant capacity and the concentration of Reactive Oxygen Metabolites (ROMs) were measured using the OXY-Adsorbent (5 μL of 1:100 diluted plasma) and d-ROMs tests (5 μL of plasma, DIACRON INTERNATIONAL, s.r.l, Italy) following the manufacturer protocol (for detailed description of these tests, see [48]). OXY adsorbent test allows quantifying the ability of the plasma antioxidant capacity to buffer massive oxidation through hypochlorous acid while the d-ROMs test measures mostly hydroperoxydes, as a marker of oxidative damage (principally on lipids). Antioxidant capacity is expressed as mM of HClO neutralised and d-ROMs as mg of H_2_O_2_ equivalent/dL. For this experiment, 60 mice and 60 zebra finches were blood sampled, and all measurements were run in duplicates. Intra-individual variation in both species based on duplicates was low (respectively CV = 2.36 ± 0.38% for the OXY test and CV = 3.06 ± 0.76% for the d-ROMs test) as well as inter-plate variation based on a standard sample repeated over plates (CV = 4.21% for OXY and 4.79% for d-ROMs test).

### Statistics

Considering our limited sample size and our repeated design, we investigated treatment and species effect on mitochondrial respiration and superoxide production by running Generalized Estimated Equations (GEE [49]) with individual identity as subject, treatment as within-subject factor, species as a fixed factor and the interaction between those two parameters (Species x Treatment). We tested differences between interaction groups using Bonferroni post-hoc procedures for GEE. We tested whether levels of respiration and superoxide production were different from zero by running one-sample t-tests, independently for each species. We investigated differences in on OXY and ROMs levels between zebra finches and mice using General Linear Models (GLM). GEE and GLMs were fitted with a normal error distribution (SPSS 18.0), and data were tested for normality and homoscedasticity. All tests were two-tailed tests and p values of less than 0.05 were considered significant. Means are quoted ± S.E.

## Competing interests

The authors declare that they have no competing interests.

## Authors’ contributions

AS designed the study. AS, PB and FC wrote the paper. AS and QS collected and analyzed the data. CR realized TEM preparations and observations. JZ, FS and BG provided support with respirometry measurements, and FG provided support with FACS measurements. SM, JZ, PB and FC took part in data analyses and interpretations. All authors have read and approved the final version of the manuscript.

## Supplementary Material

Additional file 1: Figure S1 Typical polarographic trace of respiratory activity of avian erythrocytes in response to ADP (150 μM) addition. One sample *t*-test reveals a significant change in O_2_ dynamics following ADP addition (N = 5, mean = + 4.53 nmol O_2_/min/mL; *t* = 3.20, df = 4, *p* = 0.033). ADP addition induces a strong release of oxygen, suggesting a role of ADP in the modulation of the affinity between oxygen and haemoglobin in avian erythrocytes. The methodology used is similar to the one explained in the manuscript. **Figure S2.** Mitochondrial Superoxide production in birds under normal and hyperglycaemic conditions expressed as change in mitosox red® relative fluorescence (RF) per minute. White bar represents baseline superoxide production and dashed bar represents superoxide production in hyperglycaemic conditions (incubation with 30 mM Glucose). Paired *t*-test does not reveal significant difference between treatments (N = 9; *t*_paired_ = -0.29, df = 8, *p* = 0.78). The methodology used is similar to the one explained in the manuscript. Click here for file
