# Supplementary material for: Avian erythrocytes have functional mitochondria, opening novel perspectives for birds as animal models in the study of ageing
Source: Front Zool. 2013 Jun 8;10:33. doi: 10.1186/1742-9994-10-33 (PMC3686644; doi:10.1186/1742-9994-10-33)
Supplement: Additional file 1: Figure S1 — Typical polarographic trace of respiratory activity of avian erythrocytes in response to ADP (150 μM) addition. One sample t-test reveals a significant change in O2 dynamics following ADP addition (N = 5, mean = + 4.53 nmol O2/min/mL; t = 3.20, df = 4, p = 0.033). ADP addition induces a strong release of oxygen, suggesting a role of ADP in the modulation of the affinity between oxygen and haemoglobin in avian erythrocytes. The methodology used is similar to the one explained in the manuscript. Figure S2. Mitochondrial Superoxide production in birds under normal and hyperglycaemic conditions expressed as change in mitosox red® relative fluorescence (RF) per minute. White bar represents baseline superoxide production and dashed bar represents superoxide production in hyperglycaemic conditions (incubation with 30 mM Glucose). Paired t-test does not reveal significant difference between treatments (N = 9; tpaired = -0.29, df = 8, p = 0.78). The methodology used is similar to the one explained in the manuscript. [file 1742-9994-10-33-S1.doc]

**Additional file**


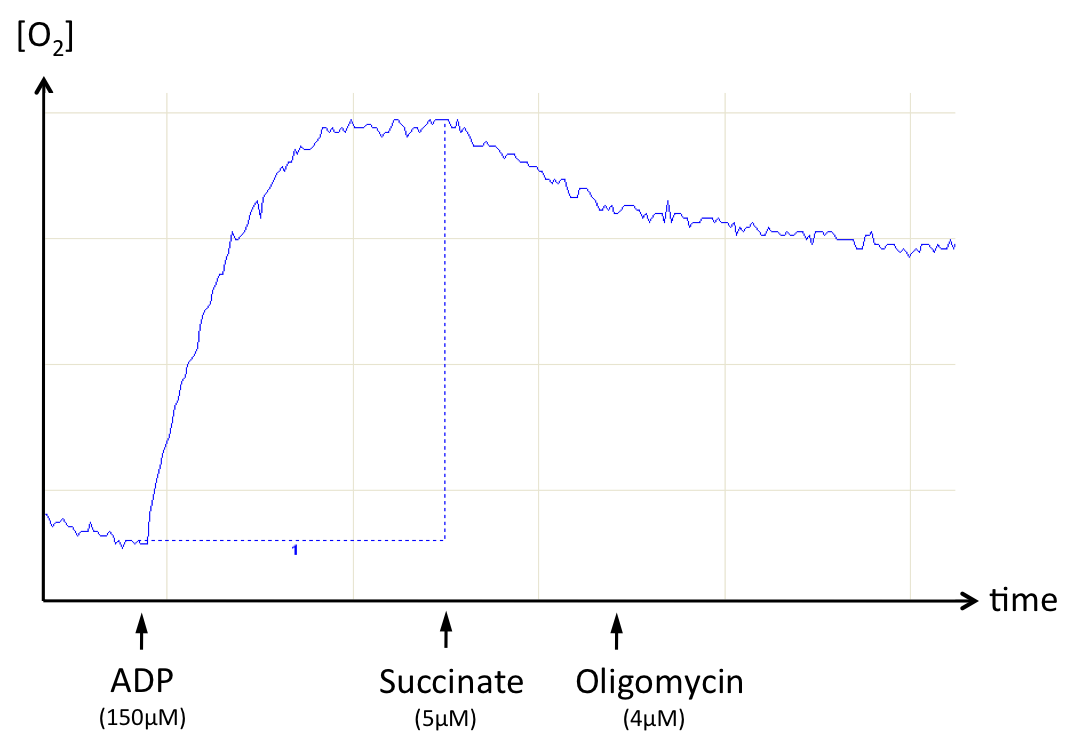


**Additional file 1: Figure S1. Typical polarographic trace of respiratory activity of avian erythrocytes in response to ADP (150μM) addition**. One sample t-test reveals a significant change in O_2_ dynamics following ADP addition (N = 5, mean = + 4.53 nmol O_2_/min/mL; *t* = 3.20, df = 4, *p* = 0.033). ADP addition induces a strong release of oxygen, suggesting a role of ADP in the modulation of the affinity between oxygen and haemoglobin in avian erythrocytes. The methodology used is similar to the one explained in the manuscript.


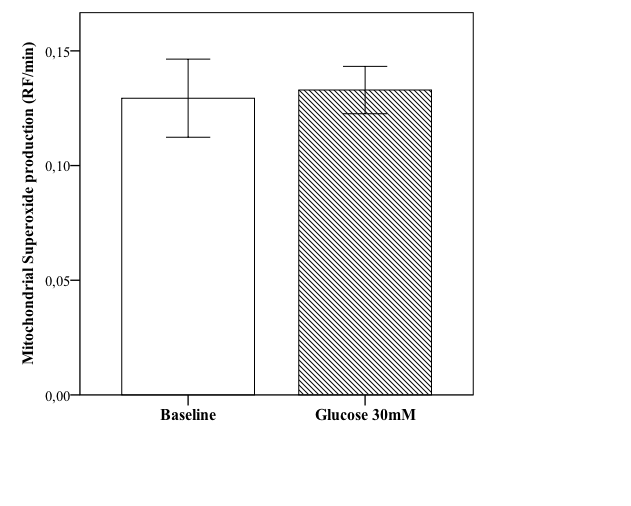


**Additional file 1: FigureS2.** **Mitochondrial Superoxide production in birds under normal and hyperglycaemic conditions** expressed as change in mitosox red® relative fluorescence (RF) per minute. White bar represents baseline superoxide production and dashed bar represents superoxide production in hyperglycaemic conditions (incubation with 30mM Glucose). Paired t-test does not reveal significant difference between treatments (N = 9; *t*_paired_ = -0.29, df = 8, *p* = 0.78). The methodology used is similar to the one explained in the manuscript.
